# Genomes of *Gardnerella* Strains Reveal an Abundance of Prophages within the Bladder Microbiome

**DOI:** 10.1371/journal.pone.0166757

**Published:** 2016-11-18

**Authors:** Kema Malki, Jason W. Shapiro, Travis K. Price, Evann E. Hilt, Krystal Thomas-White, Trina Sircar, Amy B. Rosenfeld, Gina Kuffel, Michael J. Zilliox, Alan J. Wolfe, Catherine Putonti

**Affiliations:** 1 Department of Biology, Loyola University Chicago, Chicago, Illinois, United States of America; 2 Bioinformatics Program, Loyola University Chicago, Chicago, Illinois, United States of America; 3 Department of Microbiology and Immunology, Stritch School of Medicine, Loyola University Chicago, Maywood, Illinois, United States of America; 4 Center for Biomedical Informatics, Loyola Genomics Facility, Loyola University Chicago, Maywood, Illinois, United States of America; 5 Department of Public Health Sciences, Stritch School of Medicine, Loyola University Chicago, Maywood, Illinois, United States of America; 6 Department of Computer Science, Loyola University Chicago, Chicago, Illinois, United States of America; Academia Sinica, TAIWAN

## Abstract

Bacterial surveys of the vaginal and bladder human microbiota have revealed an abundance of many similar bacterial taxa. As the bladder was once thought to be sterile, the complex interactions between microbes within the bladder have yet to be characterized. To initiate this process, we have begun sequencing isolates, including the clinically relevant genus *Gardnerella*. Herein, we present the genomic sequences of four *Gardnerella* strains isolated from the bladders of women with symptoms of urgency urinary incontinence; these are the first *Gardnerella* genomes produced from this niche. Congruent to genomic characterization of *Gardnerella* isolates from the reproductive tract, isolates from the bladder reveal a large pangenome, as well as evidence of high frequency horizontal gene transfer. Prophage gene sequences were found to be abundant amongst the strains isolated from the bladder, as well as amongst publicly available *Gardnerella* genomes from the vagina and endometrium, motivating an in depth examination of these sequences. Amongst the 39 *Gardnerella* strains examined here, there were more than 400 annotated prophage gene sequences that we could cluster into 95 homologous groups; 49 of these groups were unique to a single strain. While many of these prophages exhibited no sequence similarity to any lytic phage genome, estimation of the rate of phage acquisition suggests both vertical and horizontal acquisition. Furthermore, bioinformatic evidence indicates that prophage acquisition is ongoing within both vaginal and bladder *Gardnerella* populations. The abundance of prophage sequences within the strains examined here suggests that phages could play an important role in the species’ evolutionary history and in its interactions within the complex communities found in the female urinary and reproductive tracts.

## Background

*Gardnerella*, a member of the Bifidobacteriaceae family, is a genus of facultative anaerobes within the vaginal microbiota of both healthy women and those diagnosed with bacterial vaginosis (BV) [1–4]. Similarly, our group found *Gardnerella* in urine collected from adult female bladders by transurethral catheter [5–8]. This corresponds with microbiome studies of voided urine: *Gardnerella* was present regardless of sex or symptom status [9–13]. Furthermore, the bladders of healthy individuals include other bacterial taxa also detected within the vaginal microbiota [5–10,12–14]. To date, thirty-nine *G*. *vaginalis* isolates from the vagina or endometrium have been sequenced [15–19], including four complete genomes; the remaining genomes are represented as scaffolds or contigs. Analyses of *G*. *vaginalis* genomes found evidence of a large pangenome that consists of a modestly sized core genome in addition to strain-specific genes [15,20].

Prior investigations of *G*. *vaginalis* genomes from the reproductive tract have uncovered indications of substantial horizontal gene transfer (HGT), including the acquisition of genes from other human-associated taxa [15,20–21]. In addition to natural competence, *G*. *vaginalis* may also include phage-mediated gene transfer, as coding regions of bacteriophage (phage) origin are ubiquitous within these genomes [20]. Similarly, genomic sequences from other bacterial taxa within the vaginal microbiota also contain parts of or entire temperate phage genomes [22–23]. Prior studies have posited that lysogenic lactobacilli phages could contribute to a shift in the vaginal microbiota leading to BV (for a review, see [4]). Phages have been found to play a crucial role in the structuring of microbial communities, including those residing within the human body [24], driving bacterial genetic diversity [25] and adaptation to changes in the environment [26]. Although several phages induced from vaginal lactobacilli have been identified [27–28], currently no phages have been characterized for *Gardnerella*. While evidence suggests that phages are likely contributors to HGT in commensal communities (e.g. [22,26] and review [29]), the extent of their effect on the human microbiome is just now being explored (e.g., [30–32]). Within many other microbiota, including the bladder, the virome remains largely unexplored.

Herein, we present the genomic sequences of four *Gardnerella* strains isolated from the bladders of adult women with symptoms of urgency urinary incontinence (UUI). Comparative genomics between these strains and publicly available isolates revealed a highly conserved core genome across the bladder and vaginal isolates. Analogous to prior observations for this species, the *Gardnerella* strains isolated from the bladder also contain a large number of prophage gene sequences. The pervasiveness of prophage sequences in *Gardnerella* genomes from both the female urinary and reproductive tracts motivated our thorough bioinformatic investigation. A comprehensive interrogation of the over 400 annotated prophage gene sequences identified here provides insight into the adaptive ability of *Gardnerella*, as well as the larger community of phages within the female urinary community.

## Results and Discussion

### Genome Features

Four *Gardnerella* strains were previously isolated from the bladders of four different female patients with symptoms of urgency urinary incontinence [8,33]. While MALDI-TOF identified two of these strains as *G*. *vaginalis* (henceforth denoted as Gv18-4 and Gv23-12), it did not resolve the other two strains to the species level (G26-12 and G30-4). 16S rRNA gene sequencing for each isolate confirmed the strains belong to the genus *Gardnerella* (S1 Fig). All four isolates were sequenced, assembled, and annotated (see Methods) and are publicly available via NCBI. The genomic sequences varied slightly in size from 1.48 to 1.62 Mbp (Table 1).

**Table 1:** Genomic characteristics of four *Gardnerella* strains isolated from female bladders.

Genome	G26-12	G30-4	Gv18-4	Gv23-12
**Length**	1,484,647	1,590,395	1,624,172	1,595,255
**GC%**	42.5	41.9	40.9	41.2
**# Protein coding regions**	1172	1242	1240	1243
**# RNAs**	48	48	45	46
**# CRISPR arrays**	5	8	2	4
**# Scaffolds**	175	183	324	301
**Max Scaffold Length**	57,529	42,968	33,481	45,660
**N50 Length**	16,568	20,497	11,232	11,376
**Average Coverage**	526	714	197	631
**INSDC ID**	LWSR00000000	LXJL00000000	LWSP00000000	LWSQ00000000

**Fig 1:**
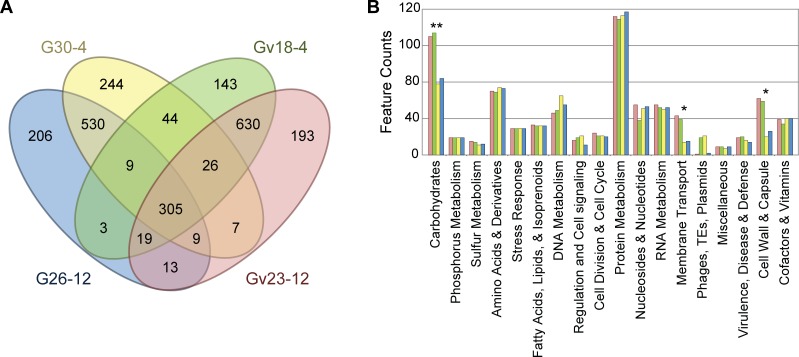
Characterization of predicted coding regions. **(A)** Homology and **(B)** annotated functionality within the four bladder *Gardnerella* strains. (**p value <0.05 between the two predicted *Gardnerella* strains and the two predicted *G*. *vaginalis* strains; *p value < 0.1). Strains are represented using the same colors in both panels.

Homologs, as well as strain-specific coding regions, were identified within the genomes of these bladder *Gardnerella* strains (Fig 1A). Although Gv18-4 and Gv23-12 were indistinguishable on the basis of their 16S rRNA sequences (S1 Fig), instances of both strain-specific gene acquisition and loss within their genomes were detected (S1 Table). Annotation also revealed the type I CRISPR/Cas system in all four genomes, with multiple CRISPR loci per genome (Table 1). Comparison between the two strains identified by MALDI-TOF analysis to the species level (Gv18-4 and Gv23-12) and those identified to the genus level (G26-12 and G30-4) revealed disparity in the number of genes with the annotated functionalities of carbohydrate metabolism, membrane transport, and cell wall and capsule synthesis (Fig 1B); however, the genetic variation observed between the Gv and G strains is statistically significant only for loci that regulate carbohydrate metabolism. Consistent with previous studies that illustrated the ability of different *Gardnerella* strains to ferment and use different carbohydrates [Harwich et al. 2010], annotation of the genome for G26-12 and G30-4 suggest that they lack coding regions with the following functionalities: chitin and N-acetylglucosamine utilization, trehalose biosynthesis, trehalose uptake and utilization, lactose and galactose uptake and utilization, lactose utilization, formaldehyde assimilation, xylose utilization, deoxyribose and deoxynucleoside catabolism, D-gluconate and ketogluconates metabolism. The absence of these coding regions within the G26-12 and G30-4 genome sequences was confirmed experimentally via PCR (see Methods).

The genomes of these bladder strains were further examined with respect to the presence/absence of coding regions associated with virulence, as previously defined by Yeoman *et al*. [20] (S2 Table). Genes associated with biofilm formation (glycosylases and glycosyltransferases) and epithelial adhesion (fimbria/pili) were identified in all four genomes; however, type-1 fimbrial precursors were found only within the genomes of G26-12 and G30-4. Genes associated with antibiotic/antimicrobial resistance, including those that encode the ABC-type multidrug transport system and the DedA protein (which has been shown to be required for drug resistance in *E*. *coli* [34]) were present within all four genomes. However, tetracycline resistance proteins were encoded within the Gv18-4, Gv23-12, and G26-12 strains, but not in the G30-4 strain. Genes associated with protection or evasion from the immune response (alkyl hydroperoxide reductase and Rib-family surface protein) were found within all four of the bladder strains. Furthermore, the coding region for the vaginolysin (VLY) gene, which is selective for human cells [20,35], was highly conserved within all four strains, in spite of the two amino acid substitutions that separate the Gv18-4 strain from the type strain for the genus ATCC 14019 (S2 Fig). Finally, the *Gardnerella* strains G26-12 and G30-4 contained an annotated rRNA methyltransferase associated with *Gardnerella* cytotoxicity [20] and with haemolytic activity in other bacterial species [36]. Interestingly, only the Gv18-4 and Gv23-12 strains contained genes that encode sialidase, which has been experimentally proven to contribute to mucin degradation in BV [16], although presence of the gene is not predictive of actual sialidase activity [37] and thus warrants further investigation.

### Phylogenetic Analysis and the *Gardnerella* Pangenome

**Fig 2:**
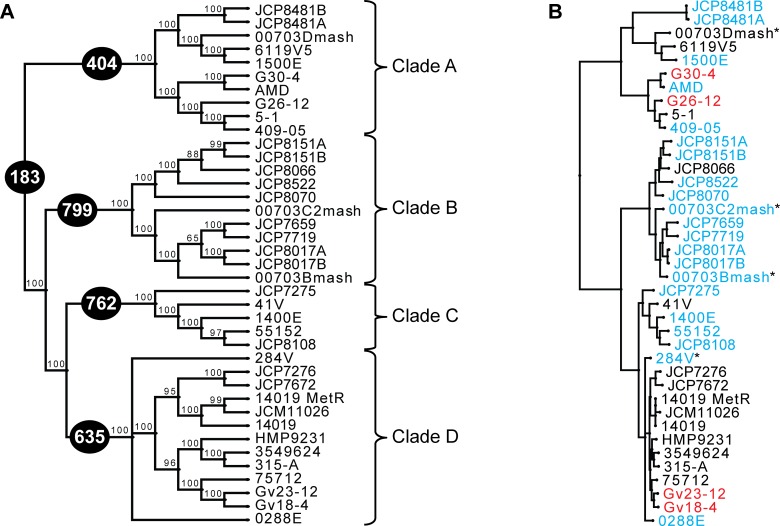
Phylogenetic Analysis of *Gardnerella* Strains. Maximum Likelihood species tree of *Gardnerella* strains based upon sequence homology within the core gene set. **(A)** Phylogenetic tree listing branch supports and distinction of the four clades within the tree. Numbers within black circles indicate the number of homologous genes within each clade and the core *Gardnerella* genome. **(B)** Maximum Likelihood tree including branch lengths and isolation information with respect to location and diagnosed symptom. Strains isolated from the bladder and sequenced in this study are labeled in red; strains listed in green were isolated from the vagina/endometrium of BV+ patients; strains in light blue were isolated the vagina/endometrium of STD+ patients. All remaining strains (indicated in black font) were isolated from the vagina or endometrium.

In an effort to assess the similarity/difference between *Gardnerella* strains isolated from the female bladder and those isolated from the female reproductive system, all publicly available sequenced strains isolated from the vagina or endometrium were retrieved from NCBI (S3 Table). This set includes 35 complete, scaffold, or contig genome sequences. Extending beyond the 16S rRNA gene marker, the evolutionary history of this genus was considered by investigating sequence homologies within the “core” *Gardnerella* genome (Methods; S4 Table). In total, 183 genes were identified as belonging to this core gene set, less than identified in prior studies of far fewer genomes [15,20]. As shown in Fig 2, the strains isolated from the bladder are not monophyletic, establishing that the *Gardnerella* core genome does not correlate with a single isolation location. Examination of all coding sequences for all 39 investigated genomes revealed no gene(s) exclusive to the bladder strains. While the vast majority of the genes within the core set demonstrated an evolutionary history identical to that of the species tree (Fig 2), genes indicative of intragenic recombination, such as VLY (S2 Fig), were also identified.

Nevertheless, the species tree (Fig 2) we derived largely concurs with the tree produced by Ahmed et al. [15]. In comparison to the phylogeny of Ahmed et al. [15], which included 17 genomes and a core genome of 473 genes, their Group 3 and 4 are both contained within Clade A in Fig 2. Our Clade B corresponds to their Group 2 and our Clade C is within their Group 1; only two of the genomes within Clade C were included in the prior analysis of Ahmed et al. [15]. As expected, each clade had significantly more homologous genes (404, 799, 762 and 635 for Clades A through D, respectively) (Fig 2A), a likely residual of undersampling, biased sampling, and/or clade-specific functional conservation.

The bladder strains G26-12 and G30-4 were determined to belong to Clade A, which also includes three strains isolated from BV+ patients (Fig 2B). While the phylogeny based upon the core gene set does not correspond with isolation location, some clades appear to have a higher incidence of strains isolated from patients diagnosed as BV+. Because genes associated with virulence are not exclusively present within genomes isolated from symptomatic patients [15,20], it is not surprising that several are included within the core gene set identified here. The lack of correspondence between phylogenetic history and symptoms is supported by prior studies, which hypothesized that gene expression variation within *G*. *vaginalis* strains may trigger BV development [38] and single point mutations may result in greater potential for cytotoxicity [39]. Thus, there appears to be no single gene that correlates with BV symptoms. This is, however, not surprising given that *G*. *vaginalis* strains are present within both the BV- and BV+ vagina [40], and it is but one of the taxa that coincides with BV symptoms [3].

### Mobile Elements

The four bladder *Gardnerella* genomes varied in their number of ORFs predicted to be viral (bacteriophage) in origin (see Methods). The number of prophage gene sequences per genome had no correspondence to evolutionary history (Fig 2). This is true for the four *Gardnerella* strains isolated from the bladder, as well as for the 35 strains isolated from the reproductive system. For example, the *G*. *vaginalis* strain JCP8522, a vaginal BV+ strain [16], has only one ORF annotated as a phage gene. In contrast, the Gv18-4 strain sequenced as part of this study contains the most, 33, phage-like gene sequences within a *Gardnerella* genome to date. S5 Table lists the 442 annotated prophage genes within the 39 genomes examined. Due to the incomplete (scaffold/contig) status of the majority of the genomes included in the analysis here, we evaluated predicted prophage genes (median length 939 bp) individually.

**Fig 3:**
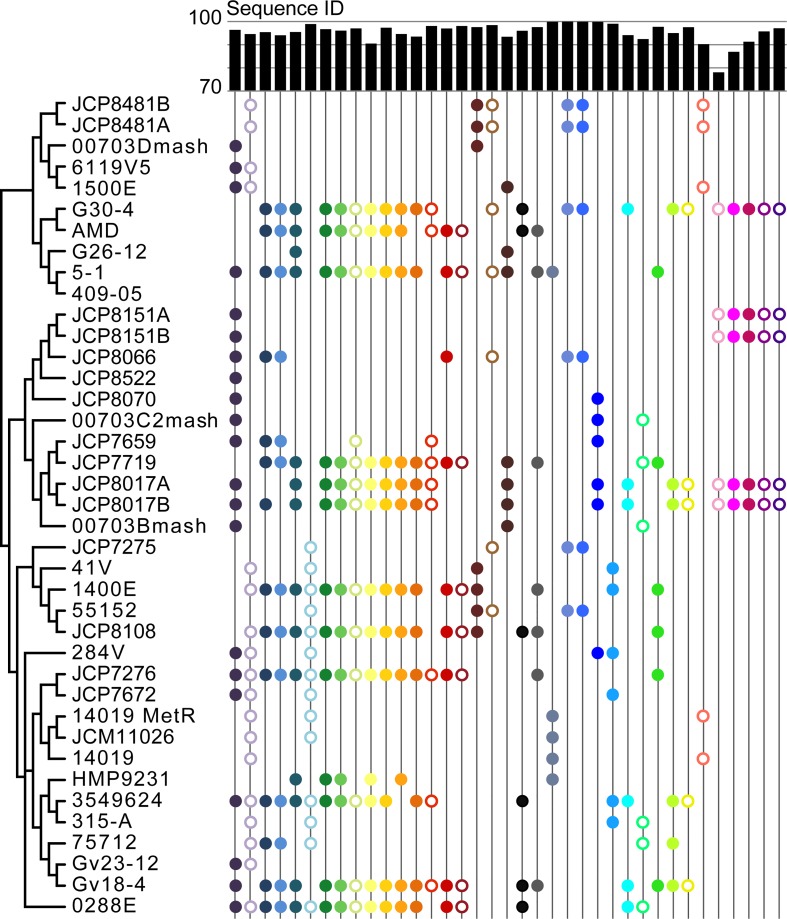
Presence/absence of prophage gene sequences from larger clusters. Clusters of orthologous prophage genes found within five or more of the 39 *Gardnerella* genomes are shown using a distinct color. Open circles indicate that the prophage sequences belonging to the particular cluster show no/poor sequence homology to characterized lytic phages. Closed circles indicate moderate (query coverage ≥80%, nucleotide sequence identity ≥35%) to high homology to a phage genome sequence record in NCBI. Nucleotide sequence identity for the prophage genes in each cluster is indicated by the bar chart at the top of the figure.

To identify homologs, the nucleotide sequences for all annotated prophage genes across all *Gardnerella* genomes were examined. Based upon the combination of sequence identity and query coverage (see Methods), 104 clusters of orthologous prophage genes were identified (S1 File). Within larger clusters, prophage homolog sequences were highly conserved between genomes. Forty-nine clusters, however, included only a single prophage gene sequence, indicative of frequent independent acquisition of viral sequences. This is further supported by the variation in prophage genes identified within strains isolated from the same patient. While the genomes of JCP8481A and JCP8481B (both isolated from a single patient as denoted by A and B [16]) contain the same set of prophage gene sequences, the genomes of JCP8151A/B and JCP8017A/B do not (Fig 3; S5 Table); this captures the likely prevalence of lysogenic phage excision, as well as gene loss, events. Likewise, the genomes of 00703Bmash, 00703C2mash, and 00703Dmash, which are from sequential isolates from the same patient [15], vary in their prophage gene content (Fig 3; S5 Table). This diversity, even within a single patient, suggests intra-host prophage gene gain/loss.

Examination of the 37 largest prophage gene clusters suggested that prophage introduction within the *Gardnerella* genus has occurred by both vertical and lateral inheritance. These larger clusters include orthologous prophage gene sequences present within five or more *Gardnerella* genomes; the most pervasive prophage gene sequences were present within 21 of the 39 *Gardnerella* genomes examined here. As shown in Fig 3, several of the larger prophage gene clusters have representatives across the entire phylogenetic tree and some sets of clusters appeared to have been lost within particular lineages. Half of the 104 prophage gene clusters identified exhibit little to no resemblance to any sequence within the current GenBank nr/nt nucleotide database of characterized phage sequences; this includes several of the prophage genes within the larger clusters (represented as open circles in Fig 3). For those prophage gene sequences that exhibit homology to characterized phage genes, hits were frequently identified to the genomes of *Bacillus*-, *Mycobacterium*- and *Staphylococcus*-infecting phages. Previous targeted gene surveys of the bladder have routinely found *Staphylococcus* within the community [6,7,33].

Although *Gardnerella*-infecting phages have yet to be isolated, the abundance of prophage gene sequences in the genomes (S5 Table) and the presence of the CRISPR/Cas-system (Table 1) suggests that phages capable of infecting *Gardnerella* spp. exist. This assumption is corroborated by prior analyses of spacer sequences within 21 *G*. *vaginalis* genomes, in which 70.7% of the spacers showed no homology to sequences within the GenBank database [21]. While many of the clusters exhibiting homology to sequences in GenBank identified lytic phages annotated as infecting an array of different bacterial genera, analysis of eleven of the 104 clusters resulted in the description of phages thought to infect a single taxon. These included several of the larger clusters shown in Fig 3: clusters 12, 15, and 30 were found to have homology to *Bacillus*-infecting phages; clusters 22 and 35 were determined to be similar to phages infecting *Mycobacterium* species; while cluster 29 exhibited a likeness to *Staphylococcus*-infecting phages (S5 Table). Yet, all of these phages are of the family *Siphoviridae*. Homologies identified for these prophage sequences provide a foundation for future work in the isolation of *Gardnerella*-specific phages. More broadly, these prophage gene sequences provide insight into the phage community within the human microbiota.

**Fig 4:**
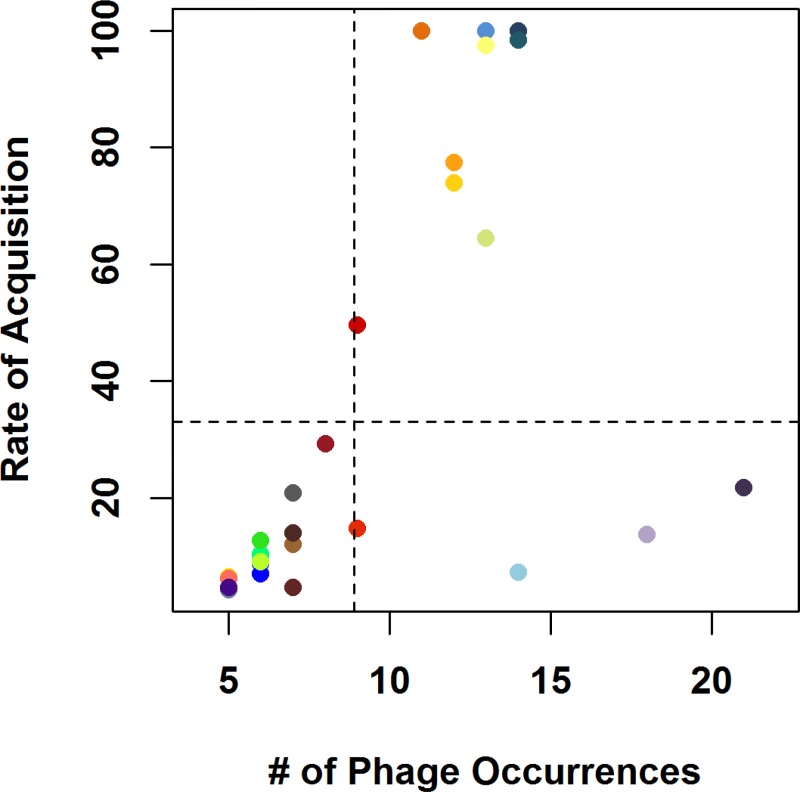
Determining mechanisms of prophage gene acquisition. Colors correspond with the cluster colors in Fig 3. Dashed lines are the mean value for each axis.

Lateral gene acquisition is ubiquitous; however, there is a strong discordance between the evolutionary tree (S1 Fig; Fig 2) and trees derived from strain:prophage gene presence/absence profiles (results not shown). To distinguish clusters acquired via HGT from those linearly inherited from a common ancestor, the genomes of 39 *Gardnerella* strains were examined. Clusters were compared given the number of occurrences and the rate of evolution of viral infection (Fig 4). From this analysis, two groups can be observed. The phage gene clusters in the top right quadrant appear to have evolved quickly relative to their prevalence across strains and have likely been integrated into the *Gardnerella* genomes via HGT. In contrast, the four clusters in the bottom right quadrant appear to have been acquired by vertical transmission. Thus, we hypothesize that prophage integration has been occurring over a long time scale. Given this evidence of phage-host interactions and the results of recent studies illuminating the vital contributions of viruses within other human microbiota (e.g. [31,32,41]), one can surmise that phages are playing a significant role within both the bladder and reproductive system communities.

As the bladder microbiota have only recently been discovered and subsequently surveyed [5–8,10,14,33,42], their microbiome remains largely uncharacterized. The presence of the CRISPR/Cas system within the four *Gardnerella* strains sequenced here suggests phages are present and prolific within the bladder. Given the existence of both *Gardnerella* and *Lactobacillus* within both the female bladder [6–8,33] and vaginal [43–45] microbiota, some insight can be gleaned from the latter. It is likely that phages play an important role in bacterial genome evolution and potentially disease in both niches. Evidence of phages has previously been found within the vaginal microbiota [27,46]. The incidence of Lactobacilliphages may have medical significance, as vaginal lactobacilli may be culled or repressed by phages within the microbiota leading to BV [27] (see review [4]). Increased prophage numbers within *L*. *crispatus* genomes from the human vagina relative to avian isolates suggests high frequency of phage within the human microbiota [23]. Phage-like sequences within both the bladder and vaginal bacterial microbiomes–prophage sequences as well as CRISPR spacer sequences–often show little to no homology to characterized sequenced phage species, insinuating that numerous genetically diverse phages have yet to be discovered.

## Conclusions

Comparison of four new bladder-associated *Gardnerella* genomes to genomes from the reproductive system identifies strain-specific and lineage-specific genes, suggesting a large *Gardnerella* pangenome may exist. There is, however, no distinct difference between strains isolated from a particular niche. Of particular interest is the high incidence of prophages within the *Gardnerella* genomes and the variability in the number per strain, as well as their putative origin. While prior studies into the prophages of vaginal lactobacilli propose that phage may play a significant role in community dynamics within the vagina (see review [4]), this proposal has yet to be empirically tested. The *Gardnerella* genome analyses conducted here find evidence of ancient, as well as contemporary, phage infection; the fact that isolates from the same individual vary in their prophage gene sequences supports the latter. Bioinformatic inspections of prophage and CRISPR spacer sequences find little to no correspondence with characterized phage sequences, suggesting that *Gardnerella*-infecting phages exist, although they have yet to be isolated in the laboratory. Nonetheless, evidence presented here suggests that phages play a role within the complex microbial communities of both the reproductive tract and the bladder, warranting future exploration of their viromes. The continued isolation and empirical characterization of *Gardnerella* species from the human microbiota is necessary to learn whether microbiome-virome interactions help to establish and maintain ones’ bladder health and, if so, how perturbations of this equilibrium result in bladder pathologies.

## Materials and Methods

### Strain Isolation and DNA Extraction

The *Gardnerella* and *Gardnerella vaginalis* isolates were isolated from transurethral catheterized urine specimens of adult women with UUI [8] using the previously described Enhanced Quantitative Urine Culture (EQUC) protocol [6]. Microbial identification was determined using a Matrix-Assisted Laser Desorption/Ionization-Time-of-Flight Mass Spectrometer (MALDI-TOF MS, Bruker Daltonics, Billerica, MA) as described [6]. Pure cultures were stored at -80°C in a 2 ml CryoSaver Brucella Broth with 10% Glycerol, no beads, Cryovial, for preservation (Hardy Diagnostics).

The preserved pure culture isolates were grown on CDC Anaerobic 5% sheep blood (Anaerobic BAP) agar (BD BBL™ Prepared Plated Media) under anaerobic conditions at 35°C for 48 hours. MALDI-TOF MS was performed for species/genus verification. An isolated colony was transferred to 5mL tryptic soy broth (TSB) supplemented with 10% fetal bovine serum (FBS) and grown under anaerobic conditions at 35°C for 48 hours. 1 mL of culture was collected, and cells were resuspended in 1 mL of buffered saline peptone (PBS).

Genomic DNA extraction was performed using a phenol-chloroform extraction protocol. Briefly, cells were resuspended in 0.5 mL DNA Extraction Buffer (20 mM Tris-Cl, 2 mM EDTA, 1.2% Triton X-100, pH 8) followed by addition of 50 μL Lysozyme (20mg/mL), 30 μL Mutanolysin, and 5 μL RNase (10 mg/mL). After a 1-hour incubation at 37°C, 80 μL 10% SDS, and 20 μL Proteinase K were added followed by a 2-hour incubation at 55°C. 210 μL of 6 M NaCl and 700 μL phenol-chloroform were then added. After a 30-minute incubation with rotation, the solutions were centrifuged at 13,500 RPM for 10 minutes, and the aqueous phase was extracted. An equivalent volume of Isopropanol was then added, and solution was centrifuged at 13,500 RPM for 10 minutes after a 10-minute incubation. The supernatant was decanted and the DNA pellet was precipitated using 600 μL 70% Ethanol.

### Genome Sequencing, Assembly, and Annotation

DNA samples were diluted in water to a concentration of 0.2 ng/μl as measured by a fluorometric-based method (Life Technologies) and 5 μl was used to obtain a total of 1 ng of input DNA. Library preparation was performed using the Nextera XT DNA Library Preparation Kit (Illumina) according to manufacturer’s instructions. The isolates were barcoded, pooled and each isolate was sequenced twice, on two separate runs, using the Illumina MiSeq platform and the MiSeq Reagent Kit v2 (300-cycles) to produce 150 bp paired-end reads. Sequencing reads were parsed into individual folders according to the respective barcodes.

The following protocol produced an assembly with the least number of scaffolds and the highest overall coverage (Table 1). Reads were paired using Geneious (Biomatters Ltd., Auckland, New Zealand) for each isolate for each sequencing run. *De novo* assembly was performed, combining the two runs per isolate, using the Geneious plug-in for Velvet [47] (*k* = 99). Sequence contigs were then extended and scaffolds were constructed using the tool SSPACE [48]. Resulting contigs were again assembled using the Geneious *de novo* assembler at the Medium-Low sensitivity setting. Annotations were performed for each of the contigs using the RAST annotation pipeline [49], as well as the BASys bacterial annotation system [50]. CRISPR arrays were predicted using CRISPRdb [51]. A local nucleotide database was created for each strain using the protein coding regions predicted by RAST. Each predicted coding region was then reciprocally BLASTed (blastn). Genes were determined to be homologs if the query coverage and the sequence identity were both greater than 70%.

Raw sequencing reads as well as assembled contigs are available through NCBI: Gv18-4 (SRA: SRX1688291, WGS: LWSP00000000), Gv23-12 (SRA: SRX1688198, WGS: LWSQ00000000), G26-12 (SRA: SRX1688301, WGS: LWSR00000000), and G30-4 (SRA: SRX1688300, WGS: LXJL00000000). S1 Table lists the annotated protein functionalities within the four strains; annotations are available and can be queried through NCBI.

### Phylogenetic Analysis

#### Single gene trees

16S rRNA gene sequence analysis was performed by excising the full length 16S sequence from the assembled genomes and querying each against the NCBI nr/nt database via blastn. Sequences producing full-length hits were collected and aligned using MUSCLE [52]. The phylogenetic tree was constructed using RAxML [53] and visualized using PhyloWidget [54]. Phylogenetic analysis of the VLY gene sequences was aligned using ClustalW [55]. As before, RAxML [53] and PhyloWidget [54] were used to derive and visualize the tree, respectively.

#### Core genome tree

While 43 strains are presently publicly available through NCBI for *G*. *vaginalis*, this study considered only those that were (1) isolated from the vagina or endometrium and (2) are documented within the literature [15–19,39]. S3 Table lists the 35 publicly available strains meeting these criteria. The species tree was derived by first identifying the core set of genes within the 39 *Gardnerella* strain sequences (genome, scaffold, or contig collections). Incomplete genomes (scaffold or contig status) were retrieved from NCBI. Their sequences were individually submitted to the RAST server and annotated [49]. For the three complete genomes, G. *vaginalis* 409–05 (NC_013721), 14019 (NC_014644), and HMP9231 (NC_017456), the ffn format files were retrieved from NCBI’s FTP site. The core gene set was determined by first creating a local nucleotide BLAST database with the coding regions annotated for the *G*. *vaginalis* 14019 strain. Annotated coding regions in other sequences were BLASTed locally (using BLASTn, e-value<10^−5^), returning the top hit only. Genes producing hits in all other 38 genomes were identified as members of the core genome. The core gene set contains 183 genes (S4 Table), a significantly smaller group than previously used when considering smaller numbers of strains [15,20]. Each core gene and its orthologs were aligned using ClustalW [55]. Alignments were concatenated producing a concatenated gene alignment (super-gene alignment) using an in-house script in R (available upon request). The Maximum-likelihood tree was derived using RAxML [53]. Trees were visualized using PhyloWidget [54].

### Assessing Presence/Absence of Coding Regions of Interest

The absence of coding regions identified with key carbohydrate metabolism functionalities within the G26-12 and G30-4 genome analyses was experimentally verified via PCR. Coding regions within the Gv18-4 genomic sequence with the following functional annotations were retrieved: (1) chitin and N-acetylglucosamine utilization, (2) deoxyribose and deoxynucleoside catabolism, (3) D-gluconate and ketogluconates metabolism, (4) formaldehyde assimilation, (5) lactose and galactose uptake and utilization, (6) trehalose biosynthesis, uptake and utilization, and (7) xylose utilization. In total, 22 genes were identified. Each was BLASTed against the nr/nt database, specifying the genus *Gardnerella*, to retrieve orthologs from other strains. All annotations within the GenBank files retrieved were manually inspected verifying similar confirmed/predicted protein functions. Primers were designed for each gene, targeting conserved regions amongst all orthologs, and obtained from Eurofins MWG Operon (Huntsville, AL). In total, 66 PCR reactions were conducted, testing each of the primer pairs against: Gv18-4 (serving as a positive control), G30-4, and nuclease-free water (serving as a negative control). All Gv18-4 reactions produced amplicons of the expected sizes. G30-4 and the negative controls did not produce amplicons. Primer sequences are listed in S6 Table.

### Prophage Identification

Genes annotated as phage or viral in origin were extracted from each of the genome sequence annotations. Local BLAST databases were used for both identifying the orthologous clusters of prophage sequences, as well as the putative origin of prophage clusters. First, a local nucleotide database was created, including all of the sequences predicted as phage from all 39 *Gardnerella* strains. Each individual putative phage coding region sequence was then BLASTed (blastn) against this local database (maximum 100 results), detecting identity to itself, as well as similarity to other sequences. Each hit was further qualified; hits with a sequence identity and query coverage greater than or equal to 80% were considered homologous. Once clusters were identified, the sequences within the cluster were aligned using ClustalW [55] and manually inspected to guarantee correct clustering (S1 File). Identification for the origin of each cluster was performed similar to above; the local database used for conducting blastn searches was the complete collection of viral RefSeq coding regions. The all.fna.tar.gz file was retrieved from ftp://ftp.ncbi.nlm.nih.gov/genomes/Viruses/. Homologs were called when query coverage was ≥80%. The sequence identity threshold was considerably lower ≥35%. A cluster was considered to show no homology to any RefSeq coding region if it did not meet both of these conditions.

### Estimating Rates of Phage Acquisition

Rates of phage acquisition were estimated using the R package corHMM [56], which implements a Hidden Markov Model to estimate the rate of evolution of traits occurring across a phylogeny. Each phage was encoded as a binary trait according to its presence/absence as visualized in Fig 3. Using this method to estimate the “rate of evolution,” therefore, provides a meaningful proxy for the rate of phage acquisition across the tree. For any given phage, the rate of acquisition is expected to scale with its total number of occurrences in the tree, and horizontally transmitted phages are expected to have faster relative rates than vertically transmitted phages (as seen in Fig 4).

## Supporting Information

S1 Fig Phylogenetic tree based on 16S rRNA gene. Maximum-Likelihood phylogenetic tree for the 16S rRNA gene. Strains isolated from the bladder are indicated in red.
(TIF)

S2 Fig Phylogenetic analysis of VLY gene. **(A)** Amino acid sequence alignment of four bladder *Gardnerella* isolates and representatives from other clades; clades are indicated to the left of each strain/isolate name. Mismatches within the alignment are highlighted (red/blue text). **(B)** Maximum-Likelihood phylogenetic tree for the VLY gene. Branch supports are indicated.
(TIF)

S1 Table Presence/absence of gene functionality within the four bladder isolates. (XLSX)

S2 Table Presence/absence of virulence genes within the four bladder isolates. (XLSX)

S3 Table List of 35 genomes retrieved from NCBI for genome comparisons. (XLSX)

S4 Table Core genes identified for the 39 *Gardnerella* genomes examined. (XLSX)

S5 Table Prophage sequences, clusters, and putative origins within the 39 *Gardnerella* genomes. (XLSX)

S6 Table PCR primers for amplification of Gv-specific carbohydrate metabolism coding regions. (XLSX)

S1 File FASTA sequences for the individual prophage gene clusters. (ZIP)
