# Supplementary figures and images for: Genomes of Gardnerella Strains Reveal an Abundance of Prophages within the Bladder Microbiome
Source: PLoS One. 2016 Nov 18;11(11):e0166757. doi: 10.1371/journal.pone.0166757 (PMC5115800; doi:10.1371/journal.pone.0166757)

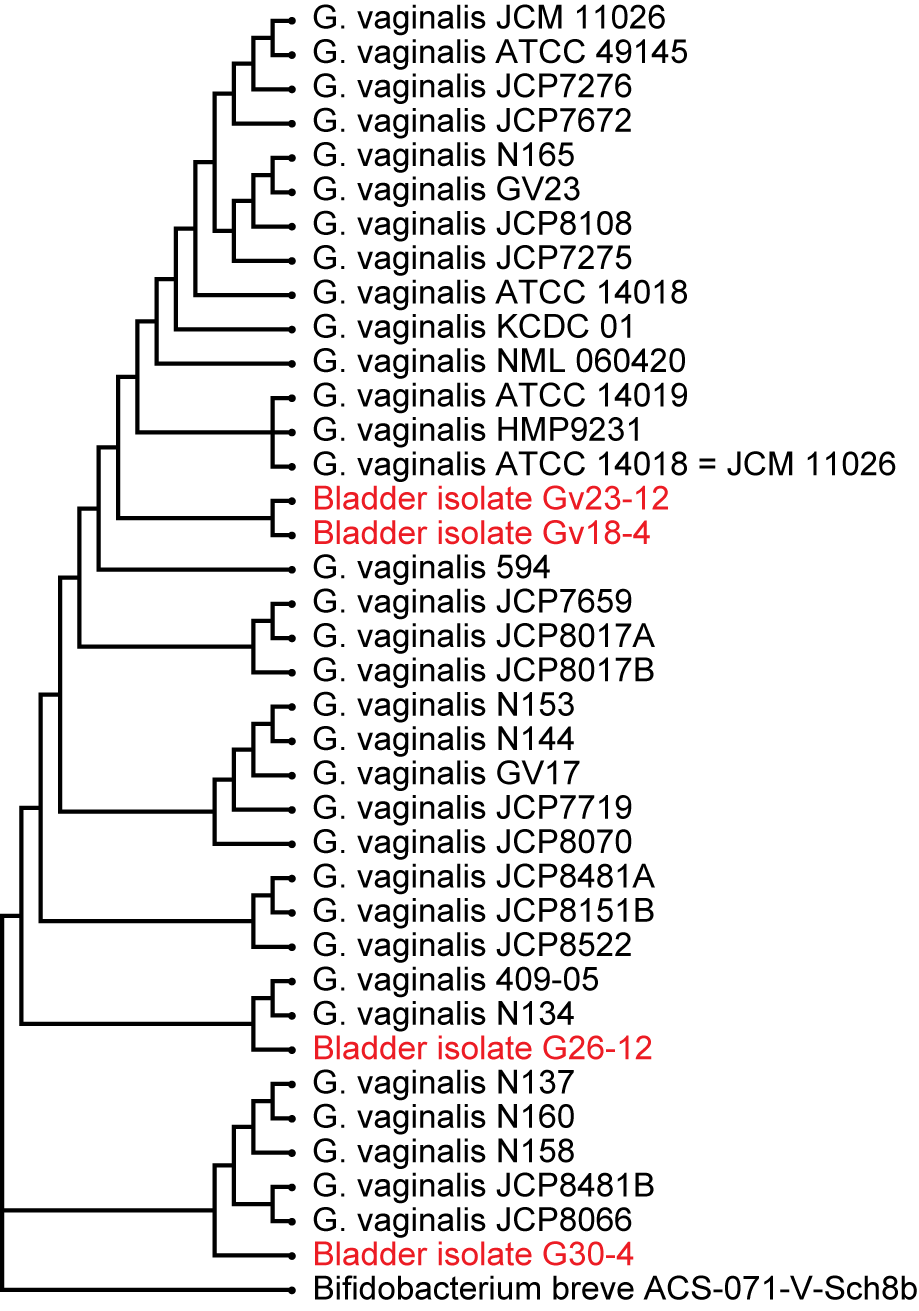

Supplement: S1 Fig — Maximum-Likelihood phylogenetic tree for the 16S rRNA gene. Strains isolated from the bladder are indicated in red. (TIF) [file pone.0166757.s001.tif]

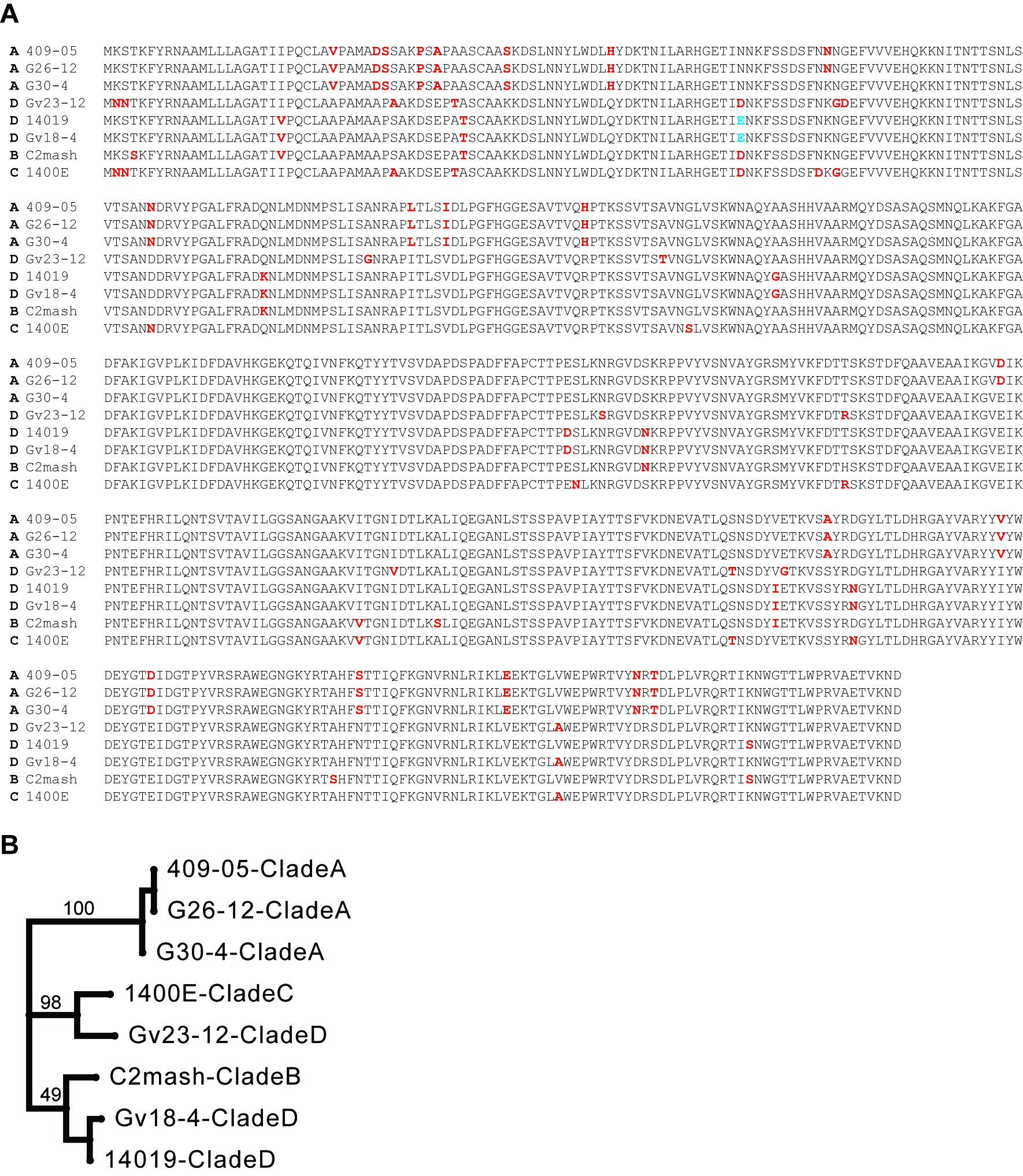

Supplement: S2 Fig — (A) Amino acid sequence alignment of four bladder Gardnerella isolates and representatives from other clades; clades are indicated to the left of each strain/isolate name. Mismatches within the alignment are highlighted (red/blue text). (B) Maximum-Likelihood phylogenetic tree for the VLY gene. Branch supports are indicated. (TIF) [file pone.0166757.s002.tif]
